# The COVID-19 pandemic and undergraduate medical student teaching/learning and assessment

**DOI:** 10.15694/mep.2021.000044.1

**Published:** 2021-02-11

**Authors:** Ian Wilson, Pathiyil Ravi Shankar

**Affiliations:** 1 International Medical University

**Keywords:** Assessment, Coronavirus disease, COVID-19, medical student, perception, teaching/learning, undergraduate

## Abstract

This article was migrated. The article was marked as recommended.

Medical colleges closed in most countries in February-March due to the coronavirus pandemic and the need to ensure physical distancing. Many rapid changes to teaching/learning and assessment were carried out. Articles published on medical education during the pandemic were analyzed to answer three questions: What changes to undergraduate medical student teaching/learning have occurred during the last six months? What modifications to undergraduate medical student assessment have occurred during the corresponding time period and what are students’ perceptions regarding these changes? English language articles published during the current year till 10 ^th^ July were searched using the terms ‘pandemic’, ‘coronavirus disease’, ‘COVID-19’, ‘undergraduate medical education’, ‘medical student assessment’ and ‘medical schools’. The online databases PubMed and Google Scholar were searched. The journal AMEE MedEdPublish was also searched. Articles dealing mainly with postgraduate education, continuing medical education, institutional preparedness, early graduation and joining the workforce were excluded.

After narrowing down according to the selection criteria, and addressing duplication, a total of 62 unique articles were obtained. A total of 44 articles were on undergraduate teaching /learning during the pandemic and 10 articles mainly focused on assessment. A total of 8 articles mentioned student experiences of teaching/learning and assessment during the pandemic. Medical education migrated online during the pandemic. The transition may have been smoother for preclinical students as the content could be delivered remotely more easily. Moving clinical learning online has greater challenges. Medical students, especially in developing countries face greater challenges to learn remotely. Open book assessments have been used. E-portfolios, projects, exams with remote proctoring, online OSCE, online structured viva-voce have been carried out. Student perception about these changes were mixed. They were happy about being able to continue learning but maintaining focus and sustaining interest were challenges. Clinical learning has been particularly affected.

## Introduction

The coronavirus disease-19 (COVID-19) pandemic caused by a novel coronavirus, now named severe acute respiratory syndrome coronavirus-2 (SARS CoV-2) has caused widespread global disruption. As of 15 ^th^ July 2020, a total of 1.3 million cases have been reported globally and 578,628 individuals have lost their lives ( COVID-19 map Johns Hopkins Corona Virus Resource Center, 2020). Most nations had closed their medical schools and colleges in February/March and some are beginning to reopen them now partially for on-site clinical learning.

A number of extremely quick modifications to teaching/learning and assessment were carried out to maintain continuity of education during the pandemic. In the absence of effective treatments and vaccines, social distancing measures have been recommended for disease control ( Sandars *et al.*, 2020). A number of articles have been published about teaching/learning in medicine, changes to assessment, and student experiences during the pandemic. In this article, the authors analyze and synthesize the published English language literature to answer three important questions. These are:

What changes to teaching/learning have happened in undergraduate medical education during the last six months?

What modifications to student assessment in undergraduate medical education have taken place during the corresponding time period?

What are undergraduate student perceptions regarding these changes?

## Methods

A review of published English language articles during the current year (2020) till 10 ^th^July 2020 was carried out using the search terms, ‘pandemic’, ‘coronavirus disease’, ‘COVID-19’, ‘undergraduate medical education’, ‘medical student assessment’ and ‘medical schools’. The databases PubMed and Google Scholar were used for the search. The online journal AMEE MedEdPublish has a large collection of articles in this area and the site was separately searched using these terms. Some of the AMEE MedEdPublish articles were also retrieved using the Google Scholar database. Majority of the articles in the COVID-19 collection are freely available online and the full text of the articles were carefully read by the authors. Articles focusing on the three questions to be addressed were included. Challenges to providing online teaching/learning and assessment were included. Studies about faculty perceptions regarding online teaching/learning and assessment were also included. Articles dealing mainly with postgraduate education, continuing medical education, institutional preparedness, early graduation and joining the workforce were excluded. We also excluded articles focusing on faculty preparation for online teaching/learning and assessment.

## Results and Discussion

Using the search criteria outlined in the Methods section a total of 146 articles were obtained. Twenty-two articles were duplicates. Twenty-four articles primarily focused on institutional preparedness and organizational matters and contribution by the institution to the surrounding community. Fifteen articles primarily focused on postgraduate and continuing education. Fourteen mainly emphasized faculty development, three dealt with conference or workshop adaptation and three addressed how to use your time effectively during the pandemic. Three articles dealt with early graduation and student perceptions about joining the workforce during the pandemic.

We obtained a total of 44 articles on undergraduate teaching /learning during the pandemic and 10 articles mainly focusing on assessment. A total of 8 articles mentioned student experiences of teaching/learning and assessment during the pandemic. Thus, a total of 62 unique articles were retrieved relevant to this study.

**Table 1.** Details of the articles included in this review

Characteristic	Number
Articles by medical students	4
*Country where the authors were working* Singapore Australia Multinational United States of America India United Kingdom Morocco Canada Italy Nigeria Mexico Nepal Malaysia Pakistan China Saudi Arabia Egypt New Zealand Turkey Ireland Bahrain Brazil	3 3 9 9 5 10 1 2 1 1 1 1 1 4 1 1 1 1 1 1 1 1
Preprints	3
*Subtopics addressed by education-related papers* Learning design Migrating to online learning Subject specific Clinical education EBM Telehealth & GP Curriculum Opportunities & challenges Impact on countries Simulation Institutional responses Peer learning Technology Narrative medicine	1 2 10 4 1 2 7 8 1 2 2 2 1
*Type of article* Original research articles Experience sharing Letters to the editor Editorial Perspective & tips	8 12 4 2 18

Four articles were entirely written by medical students. Nine articles had authors from more than one country. Authors working in the United Kingdom and the United States had the maximum number of published articles. Three articles were preprints. Subject specific papers dealt with anatomy, neuroanatomy, general surgery, otorhinolaryngology, dermatology, ophthalmology among others. Four manuscripts concentrated on clinical education in general while eight from the title of the paper focused on the impact of the pandemic on medical education in a country. Two papers detailed institutional responses (including modifications to teaching/learning and assessment) while two others focused on peer learning. There were eight original research articles mainly surveys of students and faculty, twelve were experience sharing articles either from departments or institutions while eighteen articles provided perspectives and tips.

What changes to teaching/learning have happened during undergraduate medical education?

### General considerations

Medical education has migrated online and various delivery platforms are being used. Academic medical centers should establish a COVID-19 response team which can manage various challenges associated with the pandemic in a timely manner ( Ashokka *et al.*, 2020). Among the major tasks with regard to medical education delivery are managing and coordinating remote or decentralized learning, and maintaining the integrity of formative and summative assessments while patient contact is at a minimum. For medical students during the preclinical years in developed countries the transition has been smoother as online delivery was already an important part of the curriculum ( Hilburg *et al.*, 2020). However, small group learning sessions and workshops during which students interact with faculty and peers have been impacted and been replaced by sessions using video conferencing software. A number of platforms like Zoom, Microsoft Teams, and Blue Jeans are being widely used. In some schools, this software was already being used along with learning management systems (LMS) while in many schools, especially in developing countries free versions of the software with reduced functionality are being used.

A recent article provides tips to educators to shift teaching/learning rapidly online ( Sandars *et al.*, 2020). The authors rightly mention that online learning is a rapidly growing collection of methods and technologies rather than a single, compact entity. Organizational support is required and the existing LMS should be used. Lectures should be broken down into multiple components and polls and activities are useful to maintain student interest. A flipped classroom approach is strongly recommended. If technical requirements can be met synchronous platforms are to be preferred. Asynchronous discussion forums and tutorials are also important. The optimal use of social media to communicate with students, provide feedback and reduce anxiety is important ( Sandars *et al.*, 2020). Content sharing tools, such as Hootsuite ( https://hootsuite.com/) or Buffer ( https://buffer.com/) enable educators and students to schedule and automatically post in multiple chosen platforms saving precious time. Students can be encouraged to co-create content and busy clinicians should be on board and convinced about the benefits of online learning. Similar recommendations were provided by another article which also mentions about co-creation of content, enhancing online teacher presence, delivering content as interactive modules, using flipped learning methods, providing timely feedback and supporting healthy group work ( Reyna, 2020). Students need support for online learning.

Technology plays an important role in learning and can be used for online delivery as mentioned previously. Even post-pandemic, technology will continue to play an important role in learning ( Goh and Sandars, 2020). Adaptive learning provides a personalized approach to learning for all students and informs educators how students are learning particular topics. The time for developing individual competence can be reduced. Extended reality, which includes virtual and augmented reality and haptic stimulation is also likely to play an important role. The anytime, anywhere approach can extend learning to students who may be disadvantaged. Open educational resources which are freely available to educators and students globally can reduce costs ( Goh and Sandars, 2020). Institutions, especially in low and middle income countries can face particular challenges in using technology for learning ( Cecilio-Fernandes *et al.*, 2020). Students may not have a dedicated space for learning at home and internet access could be an issue. Students may have to share resources with other family members. The use of podcasts may be a suitable approach. Lectures can be recorded and uploaded on learning portals for students to download at leisure. Asynchronous modes of delivery may also be useful.

### Response of countries and institutions to maintain teaching/learning

The biggest medical school in Mexico appears to have faced the challenge well incorporating a variety of e-learning approaches ( Fernandez-Altuna *et al.*, 2020). In Nepal online theory classes are being conducted for medical students despite various challenges and the authors conclude that half a loaf of bread (some amount of online learning) is better than none ( Atreya and Acharya, 2020). In Egypt, a survey among faculty and the educational leadership revealed a dichotomy of opinion with the leaders grading the institutional response much better compared to faculty members ( Shehata *et al.*, 2020). An editorial from India discusses various adaptations to learning during the pandemic and highlights the challenges of shifting clinical learning online ( Pattanshetti and Pattanshetti, 2020). In Italy, the authors found that online teaching may be adequate for the preclinical, basic sciences but the challenge was providing practical clinical teaching and the blurring of boundaries between work and home leading to concerns of privacy ( Bianchi, Gatto and Fabiani, 2020). In Turkey lectures were shifted online while practice courses, exams and the internship were shifted to the summer term ( Tokuç and Varol, 2020). The authors mention how our success against infectious diseases had led to a predominant focus on chronic diseases in the medical curriculum. Many of these articles with titles mentioning a country perspective, in reality provided information from a limited selection of institutions within the country. The experiences of two institutions, Newcastle University Medicine Malaysia ( Veasuvalingam and Goodson, 2020) and the School of Medicine, National University of Singapore ( Samarasekera *et al.*, 2020) provide ideas and strategies which can be adapted by other institutions. The major ideas are creating an institutional taskforce, empowering a working group with major responsibility for online teaching/learning and assessment, faculty development, preparing students for online learning, involving all stakeholders, sharing timely and relevant information and obtaining feedback from both students and faculty.

### Changes to preclinical curriculum

The majority of articles deal with the subject of anatomy. Cadaveric dissection is not being conducted and the risk of transmission of the virus through cadavers has put a stop to body donations with a consequent shortage of cadavers ( Ravi, 2020). A number of online platforms are now available for anatomy learning and there is a vigorous debate about their value and effectiveness as compared to traditional dissection ( Franchi, 2020). The author mentions that students’ fine motor skills, feelings of team work, emotional intelligence and empathy are all catalyzed in the anatomy laboratory through interactions with different individuals. The development of prosected specimens, image libraries and videos by departments are also recommended. At the University of Southampton in the UK, the SotonBrainHub website with its associated YouTube channel and Instagram account has been hosted since 2014 and is well appreciated by students ( Hall, and Border, 2020). A strength, weakness, opportunity, threat (SWOT) analysis of adaptations to Anatomy education in the UK and Ireland was carried out ( Longhurst *et al.*, 2020). To conduct online sessions and create educational material, educators, have to invest considerable amount of time learning new technology. Digitized cadaveric resources and 3-D anatomy models are being used by an increasing number of schools. There is increased collaboration between anatomy departments and an upskilling of faculty. The rapidity with which the pandemic developed created time constraints as there was not enough time to learn new technologies and create learning resources. With absence of face-to-face interaction there are challenges to the student-teacher relationship which has also been mentioned by other authors.

### Clinical teaching/learning

Due to absence of clinical exposure maintaining the quality of clinical teaching/learning has been a major challenge. Various areas have been addressed in the recent literature. In the US, ‘away’ surgical rotations in specialties not available at the student’s home institution play an important role in stimulating student interest in a residency in the subject and for programs to select suitable residents ( Boyd *et al.,* 2020). Short virtual exposures and providing more detailed information about rotations on the institutional website have been recommended. Digital clinical placements, virtual wards and similar innovations have been proposed by many authors. In a medical school in the UK, students receive a weekly set of online interactive cases which they review in detail. During a webinar with the specialty clinician the case is discussed and analyzed ( Sam *et al.,* 2020). At the University of Pennsylvania in the US, a virtual otorhinolaryngology head and neck surgery rotation was implemented consisting of interactive, live-streamed surgeries, telehealth outpatient visits, and virtual small group sessions ( Chao *et al.*, 2020). First person video technology was used in this and some other institutions so that the observer can see the field of view of the primary surgeon. Students may have been able to spend more time with the attending surgeon virtually than was possible during face-to-face sessions.

At the University of Newcastle in Australia, live streamed ward rounds were used as a replacement for traditional rounds ( Pennell *et al.* 2020). The rounds had three phases. During phase one a particular student observes the live stream carefully using a mobile, and formulates case presentations during the second phase. This is followed by the presentation phase where the student presents each patient sequentially to the faculty and a small group of students. In Hong Kong videos and written materials were used to supplement online small group sessions. Simple ophthalmic examination skills were taught and students could ask questions of the instructor using private messages ( Shih *et al.*, 2020). Teaching direct ophthalmoscopy using this method was however difficult. In New Zealand, e-learning programmes were created to replace general practice attachments during the lockdown ( Roskvist, Eggleton and Goodyear-Smith, 2020). Ten interactive e-learning cases were created which could be completed asynchronously. Microsoft teams was used during undergraduate dermatology teaching and video lectures, real time discussion and document sharing were employed (Nic and Murphy, n.d.). To take care of communication issues and provide support a specialist registrar in dermatology was allocated as a point of contact. In US medical schools, interactive platforms providing reviews of surgical anatomy, surgical procedure walkthroughs, practice test questions, and intricate patient cases are used to provide students with opportunities to practice differential diagnoses skills and improve clinical reasoning ( Ehrlich, McKenney and Elkbuli, 2020).

These methods were originally developed to supplement in-person learning which occurs in a clinical setting and not as standalone activities. Real-life clinical activities are more interactive, visual and demonstrative ( Jeyakumar *et al.*, 2020). Point of view filming which was discussed earlier was recommended using wearable filming technologies. Issues of privacy, consent, cost, feasibility and data security have to be addressed.

### Telehealth, narrative medicine and peer learning

Telehealth and telemedicine will play a more important role moving forward. The pandemic improved knowledge of evidence-based medicine among students with better knowledge of the link between modes of virus transmission and choice of preventive measures, and how knowledge of disease pathophysiology helps in choice of treatments ( Fourtassi *et al.*, 2020). In the current context simulation based medical education can be used to train students regarding proper donning and removing of personal protective equipment (PPE) and simulation sessions can be broadcast online ( Deng, Wang and Tsui, 2020). Virtual reality technology if available can strengthen simulation delivery.

Peer learning can play an important role during the pandemic. With different social isolation measures in operation, students feel lonely and vulnerable. Peer learning can teach time management, self-regulated learning, team work and group management skills. At a medical college in Saudi Arabia, students were encouraged to form pairs and small groups according to their choices ( Hamad, Iqbal, Alothri, Alghamadi, and Elhelow, 2020). Regular meetings significantly improved students’ sense of responsibility for their own learning. Improvement in problem solving potential, support to shift to newer methods of learning, and improved motivation was seen. Peer learning supported students to adapt better to technology-enhanced learning. In a UK medical school tutoring sessions by senior students were shifted online ( Roberts *et al.*, 2020). Promoting interaction between the tutor and students during online sessions was a challenge as has been mentioned in other studies and publications. The sessions were recorded for later viewing and tutors also provided real-life examples from wards to enhance student learning.

At Columbia University in the US facilitators of a narrative medicine workshop were challenged to transfer workshop online while still maintaining critical reflection by the participants as the pandemic progressed ( Iwai and Lusk, 2020). The curriculum was modified to focus on creative texts and personal well-being and the length of the workshop shortened. Most participants reacted postively though challenges with internet connection were mentioned.

To sum up many schools have been able to shift a good proportion of teaching/learning online. Shifting clinical teaching/learning online is more challenging. Different initiatives have been tried in this regard. Student engagement during sessions and learning from home are challenges. Internet connectivity, internet stability and availability of computers, laptops and tablets to access the sessions were also noted.

What modifications to student assessment have taken place?

Compared to the number of studies describing innovations in teaching/learning, those describing modifications to student assessment were less common. Some schools have shifted summative assessment to a later date after the situation improves ( Tokuç and Varol, 2020). An article highlighting the need to rethink assessment during the COVID-19 pandemic was published recently ( Sabzwari, 2020). In today’s world access to knowledge is not a problem and assessing and interpreting knowledge is important. Higher order levels of knowledge and problem-solving skills should be tested. For written examinations multiple venues may have to be created and if a single venue is to be used then social distancing requirements may require a larger space. E-portfolio can be an important method to assess the attainment of different competencies by students and older methods like viva-voce can be modified for the purpose of online assessment. Locally developed virtual patients will depict regional and local disease patterns and cultural contexts.

An assessment clock has been developed considering the five parameters which influence the utility of assessment including validity, reliability, educational impact, acceptability and cost or feasibility ( Wadi *et al.*, 2020). The clock incorporates the stakes of the exam (low or high stakes) and whether the situation is normal or an emergency condition along with the five parameters mentioned to help planners decide on the most important ones they should focus on during the crisis situation. For students at an earlier phase of the program, assessment can be carried out differently or postponed as there is no immediate impact on progression ( Fuller *et al.*, 2020). There is a learning curve for new methods of assessment which requires enough time and opportunities are available for both students and faculty to familiarize themselves. Well-designed open book tests and assignments are recommended and should use clinical cases and problems which cannot be easily solved using internet searches or textbooks. Software systems to monitor students and reduce cheating while they are giving exams at home using their own equipment have seen an increase in demand. Systems which lock down computers, require fingerprint and biometric identification, provide panoramic views of the room where the candidate is taking the exam and which track eye movements have been developed. There are ethical, privacy and other issues associated with their use. Student generated photos, voice tags and media clips have been mentioned as evidence for engagement with assessment, and peer or assessor commentary can be provided using shared documents or online spaces.

Open book assessments (OBA) testing higher order skills have been mentioned previously. Students’ perspective about OBA from King’s college, London was published ( Mathieson, Sutthakorn and Thomas, 2020). The authors were of the opinion that the summative OBAs used were a fair measure of their knowledge and the platform used allowed them to flag and annotate questions and come back later to unanswered questions. The question order was randomized and the time limit for the exam did not allow time to consult with others. Frequent OBAs may reduce the anxiety associated with exams. An online learning portfolio can monitor the technology used, faculty performance and satisfaction with online teaching, student engagement, student satisfaction, and student achievement through pre and post-test quizzes and reflections ( Alrefaie, Hassanien and Al-Hayani, 2020). During an online viva the assessor/teacher can play the role of a patient from whom the student obtains a history and mentions about relevant physical examinations.

An objective structured clinical examination (OSCE) is widely used to test clinical competence. At the National University of Singapore a high-stakes OSCE was conducted in a COVID-19 environment ( Boursicot *et al.*, 2020). Among the key principles which were applied were strict infection control and personal protection measures, segregation of participant groups, social distancing and physical separation of different cohorts, zoom facilitated briefings, wi-fi enabled data gathering and not allowing people to congregate in large groups. At a medical school in Bahrain virtual clinical assessment was conducted using an online platform ( Shehata *et al.,* 2020). Among the recommendations by the authors were to understand the available logistics, develop an assessment plan, assign a task force, build capacity and design the necessary forms, conduct mock examinations, maintain constant communication among team members during the exam, and define the process evaluation indicators. The online software has the features of pre-exam room, panels where the exam is conducted and a post-exam room. Recording capability and co-hosting features are very important. A virtual, high-stakes, summative pediatrics OSCE was conducted at the Uniformed Services University in the US using similar principles ( Lara *et al.*, 2020). The duration of time for each clinical encounter was 22 minutes, after which the examinee left the “exam room.” They then had 13 minutes to write a post encounter note while faculty and simulated patients (SPs) completed their assessments of the student. On completing the post encounter note, students were then returned to the “hallway” to read their subsequent “doorway folder”. They then proceeded into the next “exam room”. Altogether the students saw four cases.

Thus, formative quizzes, written exams using remote proctoring, open book exams, spotters, structured viva-voce and virtual OSCEs have been developed for student assessment. Assessments which are not high-stakes and during the earlier years of the course have been postponed in many locations. High-stakes OSCE has been conducted using a blended method in some institutions.

Let us now try to answer the final question.

What are student perceptions regarding these changes?

Medical students have been deployed during previous pandemics, especially the Spanish flu pandemic of 1918 and early graduation of medical students has been seen in some countries. At a Canadian medical school year 3 students helped with contact tracing, telephone helplines and supported faculty members with childcare and shopping chores ( Leong and Sarohia, 2020). Students find motivation by engaging with these volunteer duties and are concerned about adapting to different changes to the curriculum and teaching/learning. Student perceptions about the effectiveness of e-learning compared to conventional learning was studied in an Indian medical school ( Kaur *et al.*, 2020). Students rated the e-classes to be equally effective with regard to communication, contributing to skills and knowledge, offering better understanding and in grooming for a professional career. With regard to convenience, interaction level, individual learning needs and balancing of practical and theoretical knowledge students found e-learning sessions to be less effective. The student opinion was more negative at a medical college in Pakistan where over 75% of students had a negative perception of online learning and 86% felt it had little impact on their learning ( Abbasi *et al.*, 2020). Over 75% used mobiles to access the e-learning sessions. The authors mention that student perception regarding e-learning is mixed with both positive and negative opinions. In Brazil students were anxious, happy about the ability to continue their education through online methods, but only few of them enjoyed it ( Peloso *et al.*, 2020). Students opined that the learning of clinical material would be impaired and it could prolong their course. The greatest challenges they faced involved establishing a study routine due to distractions and absence of a suitable place to study at home. Learning in the absence of faculty members was another challenge and only 21% of students wanted online learning activities to continue post-quarantine.

An Indian medical student was of the opinion that learning of online skills by faculty and applying the same during student sessions played an important role in reducing the stress and anxiety faced by the student community ( Chatterjee, 2020). Issues with internet connectivity, audio and video quality and background noise were mentioned by other students and the author recommends doubt clearing sessions, virtual bedside simulation software, and methods to increase student engagement. In the UK, examinations for a large proportion of students were completed by the time lockdowns were enforced but over 75% of elective clinical placements were cancelled and the student assistantship period was also severely disrupted ( Choi *et al.*, 2020). Around 60% of students agreed that they felt less prepared for foundation year 1. Six UK medical schools changed the final year summative examination to be done remotely. The students recommend creating a robust system for standardization of online exams and clear guidance about extenuating circumstances while attempting the exam. In the US a nation-wide survey was conducted among students interested in neurosurgery to understand their concerns and educational interventions mentioned to ensure continued educational development ( Gaudix *et al*., 2020). Students shared concerns about conferences and networking opportunities, clinical experience, subinternships and clinical research experience. Virtual mentorship programs, virtual surgical workshops, webinars and virtual symposia were suggested.

An electronic survey of currently enrolled medical students at Duke-NUS medical school in Singapore was conducted early in the pandemic. The survey focused around a conceptual framework of students’ preferences for returning to the clinical setting ( Compton *et al.*, 2020). Nearly 65% of students mentioned that if possible, they would like to return to the clinical setting. The preference varied according to year of study, students perceived professional identity, self-motivation and concern about causing harm to patients and the healthcare system. In the UK, a twitter-based survey about student concerns regarding the COVID-19 pandemic was carried out following a one-hour national twitter discussion ( Huddart *et al.*, 2020). Among other concerns students mentioned that remote learning could impact their ability to develop clinical competences. There were differences in the extent to which individual medical schools involve their students in tackling the pandemic.

Thus electronic, remote learning provides students with an opportunity to continue their learning during the pandemic. But problems with staying focused on and engaged with the sessions were noted. Providing clinical learning in the absence of patient contact remains a major challenge. Virtual support and mentorship were considered useful.

Limitations: The articles included were from two databases and from the online journal, MedEdPublish. Newspaper and magazine articles dealing with these topics were not included. Preprints were included and only publications in English were considered. Only articles published on or before July 10 ^th^ were included.

## Conclusions

A number of initiatives have been carried out to sustain undergraduate medical student learning during the pandemic using different electronic platforms. The major challenge has been to sustain clinical learning in the absence of patient contact. Extended reality technology can be considered. Virtual simulation may have an important role to play. Issues of internet access, lack of electronic devices, absence of a quiet place to study or sit an exam without distractions have to be kept in mind. With regard to assessment written exams are being carried out through online platforms with an integrity pledge or remote proctoring. Open book exams have also been tried. Online structured viva-voce, and online OSCE for assessment of clinical skills have been done. Student feedback about online learning and assessment has been mixed. Developing a learning schedule and engagement and motivation were challenges.

A return to clinics and community placements can be considered under adequate personal protection and social distancing norms as soon as circumstances permit. Summative, high-stakes clinical assessments can be conducted using all necessary precautions. The pandemic can be used to highlight the importance of investing in health, epidemic control, community medicine and the urgent requirement to invest in development of newer antimicrobials. Studies on the effectiveness of online learning methods and online assessments should be conducted. Blended learning is likely to be common even after the pandemic. Greater cooperation between institutions, countries and regions is required.

## Take Home Messages

• The COVID-19 pandemic has led to the closure of medical school campuses and the shift of teaching/learning online.
• Teaching/learning has shifted online and large group and small group sessions are being conducted using online platforms.
• Virtual ward rounds, virtual surgical clinics, and online resources have been used for clinical teaching/learning.
• Open book assessments, remote written exams with or without proctoring, online OSCEs, and structured online viva-voce are among the different methods used for assessment.
• Student perception regarding these changes have been mixed.

## Notes On Contributors

**Dr P Ravi Shankar** MBBS, MD, FAIMER Fellow in Health Professions Education is a Faculty Member at the IMU Centre for Education, International Medical University, Kuala Lumpur, Malaysia.

**Prof Ian G Wilson** MBBS, FRACGP, MAssess & Eval, PhD, FAMEE is the Director of the IMU Centre for Education, International Medical University, Kuala Lumpur, Malaysia.
