# Supplementary material for: The COVID-19 pandemic and undergraduate medical student teaching/learning and assessment
Source: MedEdPublish (2016). 2021 Feb 11;10:44. [Version 1] doi: 10.15694/mep.2021.000044.1 (PMC10939615; doi:10.15694/mep.2021.000044.1)
Supplement: Peer review history

# Reviewer report: Reviewer response for version 1

**DOI:** 10.21956/mep.18846.r26862

**Published:** 2021-02-15

**Authors:** Shazia Iqbal

**Affiliations:** 1 Alfarabi College of Medicine Riyadh, KSA

This review has been migrated. The reviewer awarded 4 stars out of 5 This is a very interesting and useful review as the authors have touched time need subject for review. This is a well written and structured approach to write this manuscript. The authors pointed out several important issues to sustain undergraduate medical student learning during the pandemic.In the absence of patient contact, the significance of extending reality technology and Virtual simulation is a key point and medical educators must apply at medical schools. Although authors have highlighted the barriers (including the Issues of internet access, lack of electronic devices, absence of a quiet place to study or sit an exam without distractions), how to address these challenges would have to be elaborated especially in low resource countries. Articles mention related to Open book exams, Online structured viva-voce and online OSCE for assessment of clinical skills ideas in various set up can help medical educators to apply at a medical institution. I would strongly recommend readers to follow the take-home message.

Reviewer Expertise:

NA

No decision status is available

# Reviewer report: Reviewer response for version 1

**DOI:** 10.21956/mep.18846.r26860

**Published:** 2021-02-12

**Authors:** Sateesh Babu Arja

**Affiliations:** 1 Avalon University School of Medicine

This review has been migrated. The reviewer awarded 5 stars out of 5 I enjoyed reading this manuscript. It was very comprehensively written. The article is focused on comparing and contrasting the changes in medical education in teaching, bed-side teaching (clinical teaching), and assessments across the countries. This paper also highlighted the challenges while making those changes. The research methods are appropriate. The inclusion and exclusion criteria are adequate. The only suggestion is authors could have considered dividing the abstract into background, methods, results, and conclusion rather than without any subheadings.

Reviewer Expertise:

NA

No decision status is available

# Reviewer report: Reviewer response for version 1

**DOI:** 10.21956/mep.18846.r26861

**Published:** 2021-02-11

**Authors:** John Sandars

**Affiliations:** 1 Edge Hill University

This review has been migrated. The reviewer awarded 3 stars out of 5 This review highlights the massive curriculum changes during the first year of the COVID-19 pandemic but most importantly for me is that is also has a focus on the experiences of students. Like many reviews, it highlights more problems than potential solutions.Although there is potential use in describing the changes it would have been useful to have had a greater focus on the perceptions of all stakeholders (policy makers, educators, students, patients) to the curriculum changes, including their recommendations for improving the experience. This information is essential for making future decisions about policy and practice so that teaching and learning can be optimised during these extraordinary times.

Reviewer Expertise:

NA

No decision status is available
